# Sarcopenia is an independent risk factor for all-cause mortality rate in patients with diabetic foot ulcers

**DOI:** 10.3389/fnut.2023.1097008

**Published:** 2023-04-11

**Authors:** Qin Yang, Xia Ni, Yingxiao Zhang, Baozhen Zhu, Qinglian Zeng, Chan Yang, Jiale Shi, Chunlin Zhang, Jiahui Cai, Jinbo Hu, Qifu Li, Yingsong Jiang, Qingfeng Cheng, Chao Cheng

**Affiliations:** ^1^Department of Endocrinology, The First Affiliated Hospital of Chongqing Medical University, Chongqing, China; ^2^Department of Endocrinology, Tongxin County People's Hospital, Ningxia, China; ^3^Department of Intervention, Tongxin County People's Hospital, Ningxia, China; ^4^Department of Nephrology, University of Chinese Academy of Sciences, Chongqing General Hospital, Chongqing, China; ^5^Chongqing Diabetic Foot Disease Clinical Treatment Center, Chongqing, China; ^6^Department of Critical Care Medicine, The First Affiliated Hospital of Chongqing Medical University, Chongqing, China; ^7^Department of Critical Care Medicine, Xiangyang Central Hospital, Affiliated Hospital of Hubei University of Arts and Science, Xiangyang, China

**Keywords:** sarcopenia, diabetic foot ulcers, all-cause mortality, risk factor (RF), diabetes

## Abstract

**Objective:**

This study aimed to determine whether sarcopenia affects the all-cause mortality rate of patients with diabetic foot ulcers (DFUs).

**Research design and methods:**

The clinic-based observational study included 217 patients treated at the Department of Endocrinology, the First Affiliated Hospital of Chongqing Medical University during a 4-year period. All subjects underwent dual-energy X-ray absorptiometry to determine their body composition during hospitalization. Diagnosis of sarcopenia was based on the Baumgartner diagnostic criteria. Patients were followed up regularly by phone calls until April 1, 2019, and their survival status was recorded.Univariate and multivariate Cox risk ratio regression models were used to analyze factors influencing the all-cause mortality rate of patients with DFUs.

**Results:**

Of the 217 patients, 158 people survived (82.7%), 33 died (17.3%), and 26 were lost to follow-up. The median follow-up time was 23 (Range 11–34) months. The majority of patients were male (68.6%), with a mean age of 67.29 ± 11.14 years. The 5-year survival rate was 68.3% and 45.9% for all study patients (*n* = 217) and sarcopenia patients (*n* = 81), respectively. Multivariate Cox risk regression model showed that age (HR 1.042[95%CI:1.006, 1.078], *P* = 0.021), sarcopenia (HR 5.051[95%CI:1.968, 12.961], *P* = 0.001), and serum creatinine (HR 1.007[95%CI: 1.003, 1.010], *P* < 0.001) were independent risk factors for all-cause mortality rate of patients with DFUs. Kaplan-Meier survival curve indicated that the survival rate of patients with sarcopenia was significantly lower than non-sarcopenia patients (*P* < 0.001).

**Conclusions:**

Sarcopenia is an independent risk factor for all-cause mortality of patients with DFUs and hence an important prognostic factor for patients with DFUs. Active prevention and improvement of sarcopenia can potentially improve the survival outcomes of this patient population.

## 1. Introduction

Diabetic foot ulcers (DFUs) represent a common, complex, and costly complication of diabetes. Current evidence suggests that advanced microangiopathy and macroangiopathy enact essential roles in the pathophysiology of DFUs, leading to high morbidity and mortality rates (1). The reported probability of developing DFUs is 25% (2, 3), while 5-year mortality of up to 40%has been documented (4). Ample evidence substantiates that risk factors, such as age, gender as male, peripheral vascular disease, kidney disease, major amputation, and low hemoglobin levels play an important role in mortality from DFUs (4–6). Our previous studies demonstrated that sarcopenia is an independent risk factor to DFUs. Interestingly, it has been reported that diabetics with sarcopenia are associated with a higher incidence of foot ulcers, Wagner grade, and amputation rate than those without sarcopenia (7).

According to the European Working Group on Sarcopenia in Older People (EWGSOP), sarcopenia is defined as a syndrome characterized by progressive and generalized loss of skeletal muscle mass and strength with a risk of adverse outcomes such as physical disability, poor quality of life, and death (8–10). In this regard, a prospective 7-year study demonstrated that sarcopenia increased mortality by 2.32 times (11). Indeed, sarcopenia is closely associated with diabetes, with reports suggesting that patients with type 2 diabetes harbor a higher risk of sarcopenia than those without diabetes (12). Hitherto, few studies have been conducted to explore the relationship between sarcopenia and mortality in the diabetic population. A study from South Korea reported that sarcopenia increased the risk of death in patients who underwent diabetic foot amputation (13). In fact, sarcopenia is emerging as a further severe complication in T2DM, in addition to those already well known, such as cardiovascular diseases (14). In T2DM, the core pathophysiologic defects are insulin resistance in the muscle and in the liver, and pancreatic beta-cell dysfunction. However, it has been recognized that other factors play a relevant role in T2DM, especially accelerated lipolysis, gastrointestinal incretin hormones deficiency/resistance, hyperglucagonemia, increased glucose reabsorption, and brain insulin resistance (15). Notably, these factors often present a common trait, that is, some degree of inflammatory condition. Inflammation, indeed, appears one of the factors which links T2DM and sarcopenia (16). Although diabetes has been reported to affect the prevalence of sarcopenia, no study has explored the association between sarcopenia and mortality in patients with diabetic foot ulcer. The present study explored the associations between sarcopenia and all-cause mortality from DFUs. We hypothesized that sarcopenia might be an independent risk factor in all-cause mortality from DFUs and a predictive factor for patient prognosis.

## 2. Research design and methods

### 2.1. Study design and population

This clinic-based observational study included a total of 217 patients with DFUs who visited the Diabetic Foot Multidisciplinary Team of the department of endocrinology, The First Affiliated Hospital of Chongqing Medical University hospital from January 2014 to September 2018 and voluntarily completed the body composition assessment. All patients received standardized treatment during hospitalization and were followed up every 2 years by phone calls after discharge to record their survival and wound healing situations until April 1, 2019, or death. Twenty-six patients were lost to follow-up, 33 patients died, and 158 patients survived at the end of follow-up. The survival information of 191 patients was collected. Written informed consent was obtained from each participant, and the study was approved by the Ethics Committee of The First Affiliated Hospital of Chongqing Medical University (approval number: 2020-238).

### 2.2. Clinical data collection

Clinical baseline data of the patients included demographic characteristics, duration of diabetes and duration of DFUs, hospitalization duration, amputation history, smoking habits, cardiovascular and cerebrovascular diseases, insulin therapy, and diabetic microvascular and macrovascular complications. The physical examination included an objective assessment of clinical symptoms of diabetic peripheral neuropathy and peripheral arterial disease (PAD). PAD was defined as an ankle-brachial pressure index (ABI) less than 0.9 with supporting imaging evidence by duplex ultrasonography or angiography. Laboratory examination included the white blood cell count, hemoglobin, percentage of neutrophils, albumin, triglycerides, total cholesterol, low-density lipoprotein cholesterol (LDL-c), high-density lipoprotein cholesterol (HDL-c), glycosylated hemoglobin (HbA1c), serum creatinine, serum uric acid, and urinary microalbumin to creatinine ratio (UACR). The body mass index (BMI) was calculated by dividing weight by the square of height (Kg/m2). HbA1c was measured using borate affinity high-performance liquid chromatography (Trinity Biotech, ultra, Dublin, Ireland). Serum lipids including total cholesterol, triglyceride, HDL-c, and LDL-c were measured enzymatically by an automatic analyzer (Model 7080; Hitachi, Tokyo, Japan) with reagents purchased from Leadman Biochemistry Co. Ltd. (Beijing, China). Serum creatinine, urinary creatinine, and albumin were measured by a fully automatic biochemical analyzer (Modular DDP, Roche). The urinary micro- albuminuria to creatinine ratio (UACR) was calculated.

### 2.3. Measurement of body components and diagnosis of sarcopenia

Body composition was measured using a DXA Hologic scanner (Hologic Discovery QDR^Ⓡ^ Series, Bedford, MA, USA) by a trained technician, including the fat and muscle mass in the head, limbs, trunk, and internal organs. All standard procedures were carried out as previously described in the literature. The Hologic Whole Body DXA reference database software was used to estimate the regional and whole-body lean tissue. The diagnostic criteria for sarcopenia were based on the Baumgartner diagnostic criteria: appendicular lean mass index (ALMI)=appendicular lean mass (ALM=Arm LM + Leg LM) / height2 in kg/m2. The diagnostic criteria of sarcopenia were *ALMI* < 7.01*kg*/*m*2 and < 5.42*kg*/*m*2 in males and females, respectively (17).

### 2.4. Diagnosis and evaluation of diabetes and its complications

T2DM was diagnosed according to the diagnostic criteria for diabetes established by the World Health Organization (WHO) in 1999 (18). Chronic diabetic complications such as diabetic peripheral neuropathy, diabetic nephropathy, diabetic retinopathy, and diabetic foot disease were diagnosed using the 2012 American Diabetes Association (ADA) guidelines (18). The degree of diabetic peripheral neuropathy was assessed by the neuropathy symptom score (NSS) and neuropathy disability score (NDS). NSS was evaluated by asking patients about their experience of pain or discomfort in the legs. NDS was assessed by the Achilles reflex, vibratory sensation, temperature (cold tuning fork) sensation, 10 g monofilament proprioception, and pin-prick sensation (19). Nerve conduction velocity (NCV) was measured by EMG/Evoked Potentiometer (Type of Keypoint 9033A07, Dantec, Denmark). In addition, minor amputation was defined as amputation at the ankle joint level and below, while major amputation was defined as above the ankle joint.

### 3. Statistical analysis

Analyses were performed using SPSS 20.0 statistical software. Data were tested for normality and homogeneity of variance by a one-sample Kolmogorov-Smirnov test. Continuous variables that met the normal distribution were expressed as mean ± standard deviation. Independent samples *t*-test was used for comparison between two groups. Continuous variables that did not follow the normal distribution after data transformation were expressed as median (quartile). The comparison between groups was conducted using the two-sample Kolmogorov-Smirnov test. Categorical variables were expressed as frequencies and percentages, and the chi-square test was used for two or more groups of categorical variables. Kaplan-Meier survival curves were plotted, and the log-rank test was used to compare the survival of each group. Cox proportional hazards regression was used to obtain hazard ratio (HR) and 95% confidence interval (CI) of mortality. ăCovariates established as clinically significant predictors of death and with a *p* ≤ 0.10 during univariate analysis were entered as covariates into multivariate proportional hazards regression models for death. A *P* < 0.05 was statistically significant.

## 4. Result

### 4.1. Baseline clinical and biochemical characteristics of the study population

Table 1 shows the baseline clinical data and biochemical tests of the survival or death groups of patients with DFUs. A total of 217 individuals were included in the study, consisting predominantly of males (68.6%) with a mean age of 67.29 ± 11.14 years. The survival status of patients was followed up by telephone every 2 years, with a median follow-up time of 23 (11–34) months. As of April 1, 2019, 26 patients were lost to follow-up. Survival data were available for 191 patients, of which 158 (82.7%) survived, and 33 (17.3%) patients died. As shown in Table 1, the death group was significantly older than the survival group (*P* < 0.001). Moreover, in the death group, the serum creatinine (*P* = 0.001) was significantly higher, and with a more significant proportion of patients with a previous history of foot ulcer (*P* = 0.020) and PAD (*P* = 0.001) compared to the survival group. Hemoglobin was significantly lower in the death group than in the survival group (*P* = 0.022). Importantly, the prevalence of sarcopenia was in the death group was significantly higher than in the survival group (78.8% vs. 34.8%, *P* < 0.001). No patients included in this study underwent major amputations, and no significant difference in the history of minor amputations was found between the two groups. In addition, gender, duration of diabetes, duration of diabetic foot ulcer, smoking habits, hypertension, coronary heart disease, and history of cerebrovascular disease exhibited no significant difference between the two groups.

**Table 1:** Baseline clinical and biochemical characteristics of the survival and death group of patients with diabetic foot ulcers.

	**Alive (158,82.7%)**	**Death (33,17.3%)**	***p*-value**
Gender (male/female)	107/51	24/9	0.694
Age (year)	65.68 ± 10.69	75.00 ± 10.12	< 0.001
The hospitalization time (days)	17 (11,24)	15 (11,20)	0.240
Duration of diabetes (year)	10 (5,17)	10 (5,16)	0.985
Duration of DFUs (year)	1.0 (0.5,3.0)	0.9 (0.3,2.7)	0.206
Smoking (%)	50.6	60.6	0.297
History of foot ulcer (%)	25.3	45.5	0.020
Previous minor amputation (%)	22.8	30.3	0.358
Sarcopenia (%)	34.8	78.8	< 0.001
Hypertention (%)	61.4	57.6	0.683
Coronary heart disease (%)	18.4	27.3	0.243
Cerebrovascular disease (%)	15.8	21.3	0.451
BMI (Kg/m2)	24.55 ± 3.38	22.77 ± 3.89	0.101
White blood cell (*109/L)	8.41 ± 3.88	9.28 ± 4.61	0.262
Percentage of neutrophils (%)	71.86 ± 9.49	74.15 ± 10.59	0.220
Hemoglobin (g/L)	120.93 ± 18.58	112.39 ± 22.38	0.022
Albumin(g/L)	37 (33,41)	37 (32,39)	0.749
Total Cholesterol (mmol/L)	3.78 ± 1.08	3.58 ± 1.23	0.326
Triglyceride (mmol/L)	1.44 ± 1.51	1.23 ± 0.55	0.407
HDL-cholesterol (mmol/L)	1.04 (0.81, 1.21)	1.04 (0.86,1.23)	0.905
LDL-cholesterol (mmol/L)	2.19 (1.74, 2.94)	2.06 (1.45, 2.63)	0.446
Creatinine (umol/L)	73 (60,98)	91 (79, 157)	0.001
Uric acid (umol/L)	286.33 ± 104.25	297.67 ± 112.50	0.576
UACR (mg/g)	339.27 ± 658.01	408.71 ± 935.53	0.475
HbA1c (%)	9.87 ± 2.73	8.94 ± 2.28	0.081
ABI	1.15 (0.98 1.21)	0.88 (0.74, 1.10)	< 0.001
Insulin use (%)	53.2	54.5	0.885
**Chronic diabetic complications**			
Diabetic peripheral neuropathy (%)	94.3	87.9	0.183
Diabetic kidney disease (%)	50.0	51.5	0.874
Diabetic retinopathy (%)	40.1	37.5	0.782
Peripheral artery disease (%)	19.7	52.2	0.001

DFU is associated with Wagner grading.

### 4.2. Cumulative survival rates at 1, 3, and 5 years of follow-up for patients with diabetic foot ulcers

The cumulative survival rates of all patients at 1, 3, and 5 years of follow-up are presented in Table 2. The cumulative survival rate of diabetic foot ulcer patients with sarcopenia was 83.7% (75.3–92.1%) at 1 year and 45.9% (27.5–64.3%) at 5 years. Moreover, the cumulative survival rates at 1, 3, and 5 years were higher in patients with diabetic foot ulcers without sarcopenia than in patients with sarcopenia.

**Table 2:** Cumulative probabilities (with 95% CI) of survival.

	**Year 1**	**Year 3**	**Year 5**
All patients	92.1% (88.2–96.0%)	77.4% (69.4–85.4%)	68.3% (57.3–79.3%)
Patients with sarcopenia	83.7% (75.3–92.1%)	61.2% (46.9–75.5%)	45.9% (27.5–64.3%)
Patients without sarcopenia	98.2% (95.7–100.0%)	89.4% (80.8–98.0%)	85.4% (74.0–96.8%)

### 4.3. Prognostic factors for all-cause mortality rate in patients with DFUs (univariate and multivariate analyses)

**Figure 1:**
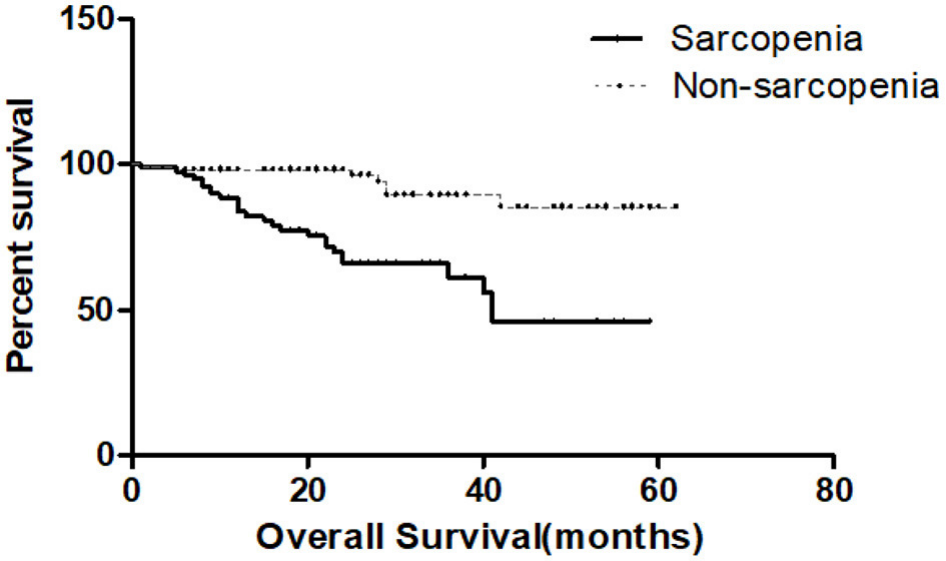
Kaplan-Meier survival plot for all subjects.

Table 3 shows the Cox risk ratio model results of all-cause mortality during univariate and multivariate analysis in patients with DFUs. During univariate analysis, factors associated with higher mortality encompassed age, sarcopenia, hemoglobin, serum creatinine, and peripheral artery disease. Gender, hospitalization time, smoking, minor amputation, previous diabetes foot ulcers, Duration of Diabetes and DFUs, HbA1c, UACR, inflammatory markers level, blood lipids, insulin use, diabetic peripheral neuropathy, diabetic nephropathy, and diabetic retinopathy (partial data not shown) exhibited no significant associations with all-cause mortality of patients with DFUs. Indexes with a *P* < 0.1 in Table 1 were included in Model 1 during multivariate analysis. We found that age, sarcopenia, hemoglobin, and serum creatinine were correlated with all-cause mortality of patients with DFUs. ăGiven the clinical impact of chronic diseases on all-cause mortality, gender, hypertension, coronary heart disease, cerebrovascular disease, diabetic nephropathy, and statistically significant indicators from Model 1 were included in Model 2 during multivariate analysis. We found that age (HR 1.042[95% CI: 1.006, 1.078], *P* = 0.021), sarcopenia (HR 5.051[95% CI: 1.968, 12.961], *P* = 0.001), and serum creatinine (HR 1.007[95% CI: 1.006, 1.078], *P* = 0.021). 01.003, 1.010], *P* < 0.001) remained significantly correlated to all-cause mortality (Table 3). Kaplan-Meier survival curves of DFUs patients in Figure 1 with and without sarcopenia showed that the survival rate of DFUs patients with sarcopenia was lower than those without sarcopenia (*P* < 0.001).

**Table 3:** Prognostic factors for all-cause mortality rate in patients with DFUs (univariate and multivariate analyses).

**Prognostic factor**	**Univariate**		**Multivariate (Model 1)**		**Multivariate (Model 2)**	
	***P*** **-value**	**HR (95% CI)**	***P*** **-value**	**HR (95% CI)**	***P*** **-value**	**HR (95% CI)**
Age	< 0.001	1.062 (1.029, 1.096)	0.026	1.064 (1.007, 1.123)	0.021	1.042 (1.006, 1.078)
History of foot ulcer	0.153	1.657 (0.829,3.309)	0.781	0.864 (0.309, 2.421)	-	-
Sarcopenia	< 0.001	6.106 (2.646, 14.091)	0.003	5.411 (1.803, 16.242)	0.001	5.051 (1.968, 12.961)
Hemoglobin	0.016	0.979 (0.962, 0.996)	0.042	0.972 (0.945, 0.999)	0.242	0.988 (0.967, 1.008)
Creatinine	< 0.001	1.006 (1.004, 1.009)	< 0.001	1.007 (1.003, 1.010)	< 0.001	1.007 (1.003, 1.010)
HbA1c	0.109	0.885 (0.761, 1.028)	0.512	1.069 (0.876, 1.304)	-	-
Peripheral artery disease	0.002	3.536 (1.559, 8.020)	0.914	0.942 (0.321, 2.770)	-	-

Notes:Model 1: Adjusted for age, history of foot ulcer, sarcopenia, hemoglobin, creatinine, HbA1c and peripheral artery disease; Model 2: Adjusted for age, sarcopenia, hemoglobin, creatinine, gender, hypertension, coronary heart disease, cerebrovascular disease and diabetic nephropathy. Abbreviations:HR, Hazard Ratio; 95% CI, 95% confidence intervals in brackets; HbA1c, glycosylated hemoglobin.

### 4.4. Prevalence of chronic diseases among DFUs patients with and without sarcopenia

As shown in Table 4, gender (*P* = 0.003) and age (*P* = 0.006) exhibited statistically significant differences between the sarcopenia and non-sarcopenia groups. The proportion of males with sarcopenia was significantly higher. Moreover, the mortality rate in the sarcopenia group was significantly higher than in the non-sarcopenia group (32.1% vs. 6.4%, *P* < 0.001). It is worth noting that the prevalence rate of chronic diseases was significantly higher in the sarcopenia group, where the prevalence rates of cerebrovascular disease, coronary heart disease, peripheral artery disease, and diabetic nephropathy were higher than in the non-sarcopenia group.

**Table 4:** Chronic disease in DFU patients with and without sarcopenia.

	**Alive (81, 42.4%)**	**Death (110, 57.6%)**	***p*-value**
Gender (male/female)	65/16	66/44	0.003
Age (year)	69.86 ± 11.23	65.39 ± 10.73	0.006
Death (%)	32.1	6.4	< 0.001
History of foot ulcer (%)	32.1	26.4	0.387
Previous minor amputation (%)	25.9	22.7	0.609
Hypertension (%)	66.7	56.4	0.150
Cerebrovascular disease (%)	24.7	10.9	0.012
Coronary heart disease (%)	27.2	14.5	0.031
Peripheral artery disease (%)	52.2	19.7	0.001
Diabetic Kidney Disease (%)	60.5	42.7	0.015

## 5. Discussion

In this clinic-based study on the long-term outcomes of patients with DFUs, an association between sarcopenia and all-cause mortality was documented. To the best of our knowledge, few studies have investigated the association between sarcopenia and mortality in patients with DFUs. Importantly, this study showed that sarcopenia was an independent risk factor for all-cause mortality in DFUs patients, suggesting that sarcopenia may be a significant predictor of prognosis in this patient population. Aggressive prevention and improvement of sarcopenia may improve survival in this patient population.

The molecular mechanism of sarcopenia associated with DFD has not been thoroughly investigated. According to previous reports, several possible mechanisms could explain this association. First, skeletal muscle is considered to be an endocrine organ. Muscle factors and muscle metabolites secreted by skeletal muscle mediate the communication between muscles and other organs (20). Patients with sarcopenia have altered myocyte production in their muscles (20), which may be related to sarcopenia and DFD. Second, muscle weakness is associated with a higher risk of foot injury (21), which is a common cause of DFD. Third, myopenia and DFD share similar underlying mechanisms, including oxidative stress, chronic inflammation, and mitochondrial dysfunction (22–24). Muscle overproduction of reactive oxygen and nitrogen species is observed in sarcopenia, and the risk of sarcopenia is greatly reduced by specific inhibition of oxidative stress by muscle (25, 26). Observational and biopsy studies have strengthened the link between chronic low-grade inflammation and muscular atrophy (27). Mitochondrial dysfunction in skeletal muscle has been implicated in the pathogenesis of sarcopenia, and improving the quality control of mitochondria has been considered as a potential intervention for the management of sarcopenia11. In addition, the regenerative capacity of skeletal muscle is reduced in sarcopenia, andthe decline in stem cell regeneration is well recognized in sarcopenia (28, 29). Overproduction of reactive oxygen species and nitrogen species in sarcopenia may mediate the progression of neuropathy and vasculopathy and correlate sarcopenia with DFD. The main strength of our study is the relatively large sample size of DXA-based body composition measurements. Since sarcopenia is associated with DFD and DFD patients with sarcopenia have a poor prognosis.

An increasing body of evidence suggests that DFUs represent a marker of high mortality in diabetic patients (30–32). Walsh JW et al. reported a 5-year mortality rate of approximately 50% in patients who developed DFUs (33). The overall 5-year survival rate of all patients with DFUs in this study was 68.3% and 45.9% for sarcopenia patients with DFUs. Serum creatinine, sarcopenia, and age were independent predictors of mortality in patients with DFUs after adjusting for multiple confounding factors. Hemoglobin and peripheral arterial disease were associated with DFU mortality during univariate analysis. A low hemoglobin often reflects a poor nutritional status and may be related to a poor patient prognosis. Moreover, it has been reported that peripheral vascular disease is a predictor of death for patients with DFU (34). However, the above findings were not observed after adjusting for confounding factors in this study. In addition, we found that serum creatinine is an independent risk factor for all-cause mortality of patients with DFUs. The degree of kidney damage has been documented to be closely related to the incidence and prevalence of DFU (35). In this respect, Wolf et al. reported that impaired renal function is an independent predictor of all-cause mortality and cardiovascular mortality (36). Moreover, lower limb amputation has been strongly associated with DFUs mortality and is reportedly an independent predictor of death (5, 37). There was no correlation between amputation and all-cause mortality of DFUs in this study, which may be attributed to the small number of DFUs patients with a history of amputation included in this study, and all amputation cases were minor.

It is well-established that sarcopenia is a strong predictive factor of all-cause mortality among community seniors (38, 39), nursing home residents (40), and hospitalized seniors (41). Interestingly, Atkins et al. reported that sarcopenia might be associated with cardiovascular mortality (42). Another long-term follow-up study of 15,000 Chinese middle-aged and elderly people showed that compared with normal people, the incidence of cardiovascular disease in middle-aged and elderly people with sarcopenia increased by 72%, and the risk of cardiovascular events increased by 33% (43). Consistently, the present study indicated that sarcopenia was an independent risk factor for all-cause mortality in patients with DFUs. As shown in Table 4, significant differences in gender and age were found between sarcopenia and non-sarcopenia patients. Importantly, the prevalence rates of diabetic nephropathy, peripheral artery disease, coronary heart disease, and cerebrovascular disease were higher among patients with sarcopenia, accounting for the significant impact of sarcopenia on mortality of patients with DFUs.

Several limitations and shortcomings were found in this study. First of all, only patients with DFUs who voluntarily completed the body composition exam were included, representing a source of selection bias. For clinical reasons, some severe patients with DFUs who were unable to move freely failed to complete body composition tests during hospitalization, but clinical observations found that these patients often had sarcopenia, which may allow us to underestimate the prevalence of sarcopenia in patients with DFUs. This is not conducive to our discovery of a possible closer association between sarcopenia and all-cause mortality in patients with DFUs. Moreover, the sample size of this study was relatively small, and the median follow-up time was short, which may lead to biased results. In addition, all amputation cases in this study were minor, which may be attributed to the fact that patients with major amputations exhibit poor ambulation and cannot complete the examination's physical component. Accordingly, we could not properly explore the relationship between mortality and major and minor amputations in sarcopenia patients. For patients lost to follow-up, it is highly likely that some patients were already deceased and could be contacted, which led to an underestimation of the actual mortality rate. Moreover, the specific cause of death could not be ascertained during telephone follow-up. Accordingly, this study only explored the association between sarcopenia and all-cause mortality of patients with DFUs. In addition, no data was available on muscle strength and muscle function, parameters emphasized in the diagnostic criteria of sarcopenia in recent years. Therefore, prospective follow-up studies with a larger sample size are warranted to increase the robustness of our findings and explore the associations between muscle strength and function and prognosis of this patient population.

In summary, sarcopenia is an independent risk factor for all-cause mortality of patients with DFUs, suggesting it is an important prognostic factor. Active prevention and improvement of sarcopenia may increase the survival rates of this patient population.

## Data availability statement

The original contributions presented in the study are included in the article/supplementary material, further inquiries can be directed to the corresponding authors.

## Ethics statement

The studies involving human participants were reviewed and approved by the Ethics Committee of the First Affiliated Hospital of Chongqing Medical University (approval number: 2020-238). The patients/participants provided their written informed consent to participate in this study.

## Author contributions

QC and QY designed the study. YZ, BZ, QZ, CY, JS, CZ, XN, and JC collected and collated clinical data. JH and YJ take responsibility for the accuracy of the data analysis. QY and XN drafted the manuscript. QC was the guarantor of this work and, as such, had full access to all the data in the study and take responsibility for the integrity of the data and the accuracy of the data analysis. All authors provided support for the analysis and interpretation of results, critically revised the manuscript, and approved the final manuscript.
